# A Portable Sign Language Collection and Translation Platform with Smart Watches Using a BLSTM-Based Multi-Feature Framework

**DOI:** 10.3390/mi13020333

**Published:** 2022-02-20

**Authors:** Zhenxing Zhou, Vincent W. L. Tam, Edmund Y. Lam

**Affiliations:** Department of Electrical and Electronic Engineering, The University of Hong Kong, Pokfulam, Hong Kong, China; vtam@eee.hku.hk (V.W.L.T.); elam@eee.hku.hk (E.Y.L.)

**Keywords:** continuous sign language recognition, gesture-based sign language recognition, smart watch, multi-feature framework, bidirectional long short-term memory

## Abstract

Continuous sign language recognition (CSLR) using different types of sensors to precisely recognize sign language in real time is a very challenging but important research direction in sensor technology. Many previous methods are vision-based, with computationally intensive algorithms to process a large number of image/video frames possibly contaminated with noises, which can result in a large translation delay. On the other hand, gesture-based CSLR relying on hand movement data captured on wearable devices may require less computation resources and translation time. Thus, it is more efficient to provide instant translation during real-world communication. However, the insufficient amount of information provided by the wearable sensors often affect the overall performance of this system. To tackle this issue, we propose a bidirectional long short-term memory (BLSTM)-based multi-feature framework for conducting gesture-based CSLR precisely with two smart watches. In this framework, multiple sets of input features are extracted from the collected gesture data to provide a diverse spectrum of valuable information to the underlying BLSTM model for CSLR. To demonstrate the effectiveness of the proposed framework, we test it on an extremely challenging and radically new dataset of Hong Kong sign language (HKSL), in which hand movement data are collected from 6 individual signers for 50 different sentences. The experimental results reveal that the proposed framework attains a much lower word error rate compared with other existing machine learning or deep learning approaches for gesture-based CSLR. Based on this framework, we further propose a portable sign language collection and translation platform, which can simplify the procedure of collecting gesture-based sign language dataset and recognize sign language through smart watch data in real time, in order to break the communication barrier for the sign language users.

## 1. Introduction

Sign language using hand gestures and body movements for transferring information is widely used among the deaf. However, sign languages are usually distinct from spoken languages in their linguistic rules; for example, American sign language is not a manual form of English [1]. It is difficult for hearing people to understand sign language without professional training, which builds a strong communication barrier between the sign language users and hearing people. To break down this communication barrier, sign language recognition (SLR) has become a potential topic in different research fields such as computer vision, sensor technology, and accessible computing.

In general, there are mainly two branches in SLR: isolated sign language recognition and continuous sign language recognition (CSLR). By definition, isolated SLR takes one word or one phase as its ground truth label, while CSLR attempts to decipher whole sentences performed by signers. CSLR is much more complicated than isolated SLR, as it not merely contains multiple words in each sample, but is confused by the co-articulation effect (the fact that the ending of the previous sign may influence the start of the following sign), as well as non-uniform speed [1]. In spite of its complexity, CSLR has greater practical significance than isolated SLR, as most people prefer sentence-level translations during daily communication [2,3]. Thereby, we target CSLR in this paper, in which sentences of sign language are translated into spoken language.

Aside from the recognition content, there are also two directions in terms of the recognition methods: vision-based SLR and gesture-based SLR. Vision-based SLR concentrates on recognizing sign language from images or videos [4,5], while gesture-based SLR relies on a gestural signal collected from different types of sensors, such as smart watches and gloves [6,7], to recognize the sign language.

In recent years, the rapid growth of computer vision technology has caused vision-based SLR to be the dominant research direction. Many researchers have contributed their efforts in finding the most effective network structures for vision-based SLR [8,9,10]. Nevertheless, despite its fast development, vision-based SLR is still hindered from real-world applicability by two vital limitations. On the one hand, most of the network structures used in vision-based SLR are quite “deep”, containing many layers between the input and the output to ensure recognition accuracy [11,12,13]. It is thus extremely challenging to deploy those network structures on edge devices and mobile phones with limited computation resources. On the other hand, generalization to various types of environmental conditions has been one of the most challenging issues since the birth of computer vision [14,15], and vision-based SLR is no exception. Bad brightness levels or irrelevant backgrounds may reduce the accuracy of vision-based SLR significantly, which seriously impedes its development. Although some advanced deep learning networks such as I3D [16] have been developed to minimize the impact of the environmental conditions, the large running delay of these networks may restrict the application value of research in vision-based SLR.

On the contrary, as it does not suffer from any of the above weaknesses related to computer vision, gesture-based SLR has gradually become one of the most promising approaches in SLR, in which sensor-intensive gloves [17,18,19], clothing [20], and watches [21,22,23] are used to record the movements of the signers. Among these devices, smart watches are one of the most popular choices for SLR, as they are not only available from the market, but also minimize intrusiveness to the signers. Thus, we adopt two smart watches to conduct gesture-based SLR in this paper. However, compared with sensor-intensive gloves, smart watches can only provide two types of activity-related data—accelerometer data and gyroscope data—which increases the difficulty of conducting gesture-based SLR, since the data provided to the recognition model are limited.

To overcome this challenge, we propose a pioneering bidirectional long short-term memory (BLSTM)-based multi-feature framework for conducting gesture-based CSLR accurately. In this framework, three types of features are extracted from the smart watch data, including time domain features, frequency domain features, and convolutional neural network (CNN)-based features. All the extracted features are then concatenated and fed into the next BLSTM layer to consider the temporal dependencies between them. After that, a fully-connected layer with a softmax layer is employed to project the output of the BLSTM layer from feature space into vocabulary space and produce the results.

To evaluate the performance of this framework, we collected a new gesture-based continuous Hong Kong sign language (HKSL) dataset, in which the hand movements of 50 sign language sentences performed by six signers were recorded by smart watches. The experiment conducted on this dataset demonstrated the effectiveness of the proposed BSLTM-based multi-feature framework. Based on this framework, we further propose a portable sign language collection and translation platform for facilitating communication between sign language users and the others.

In summary, the main contributions of this work are:

1. A BLSTM-based multi-feature framework is proposed for conducting CSLR with smart watch data. In this framework, three types of features are extracted from the raw data and processed by the BLSTM layer to produce the results;
2. A portable sign language collection and translation platform was developed. This platform not only simplifies the operation of collecting gesture-based sign language datasets, but also supports both offline and online sign language translation;
3. A new gesture-based continuous HKSL dataset was collected, in which there are 50 sign language sentences performed by 6 signers with 8 repetitions. In this dataset, the accelerometer data and gyroscope data of the signers were recorded by smart watches with a sample rate of 50 Hz. This dataset will be available to the public to facilitate research into gesture-based CSLR;
4. Intensive experiments were conducted to compare the performance of different machine learning and deep learning approaches with the BLSTM-based multi-feature framework in gesture-based CSLR.

The rest of this paper is organized as follows. Some related works in gesture-based SLR will be discussed in Section 2. In Section 3, the structure of the proposed BLSTM-based and multi-feature framework will be introduced in detail. In Section 4, the newly collected HKSL dataset and the experimental results of the BLSTM-based multi-feature framework on this dataset will be presented. In Section 5, the proposed portable sign language collection and translation platform will be described. Lastly, concluding remarks and future directions will be considered in Section 6.

## 2. Related Work

To facilitate the interaction between the deaf and hearing people, significant research has been conducted on applying different types of sensor technologies in gesture-based SLR. The first work in this field dates back to 1983, in which Grimes [24] used an electronic glove for recognizing finger-spellings. Since then, research has been conducted on applying different approaches and different devices in gesture-based SLR. In 2017, Ekiz et al. [25] firstly attempted to capture the hand movements of signers with smart watches and used dynamic time warping (DTW) to compute the distances between the gestures and the templates in different dimensions for SLR.

In 2018, Kishore et al. [26] proposed a two-phase matching algorithm for isolated SLR with gloves and cameras in which they extracted the motion joints from signers and used a kernel matching algorithm to find the most likely sign in their database according to these motion joints. In 2018 as well, Lee et al. [27] designed a new wearable hand device for isolated sign language recognition in which there are five flex-sensors, two pressure sensors, and a three-axis inertial motion sensor. However, rather than using a matching algorithm, Lee et al. adopted a support vector machine (SVM) for classifying different signs.

In 2019, Deriche et al. [28] utilized leap motions for SLR, and they performed the classification through two approaches: a Bayesian approach with a Gaussian mixture model, and a linear discriminant analysis approach. Similarly, in 2019, Kumar et al. [29] applied leap motions in sign language recognition. To achieve a high recognition accuracy, they adopted a modified LSTM model with an extra RESET gate in their work. Later in the same year, Hou et al. [30] proposed a new SignSpeaker system, in which they extracted the frequency domain features from smart watch data and fed them into to next LSTM layer for SLR. Instead of using any smart watches, Yu et al. [31] attached three types of sensors, including surface electromyography, accelerometers, and gyroscopes, onto the signers to collect their data when performing isolated sign language. After that, they applied a deep belief net to conduct SLR.

In 2020, Pan et al. [32] developed a wireless multi-channel capacitive sensor for recognizing numbers from 1 to 9. In their proposed system, code-modulated signals are directly processed without any demodulation. A faster response time was thus achieved. Similarly, using capacitive sensors, Wong et al. [33] also proposed a capacitance-based glove to measure capacitance values from the electrodes placed on finger phalanges for sign language recognition. Based on this device, they extracted 15 features from the capacitive signals and compared the performance of support vector machine (SVM) with k-nearest neighbor (KNN) in classifying different alphabets according to these features.

In 2021, Ramalingame et al. [34] developed a wearable band integrated with nano-composite pressure sensors. The sensors in their work consisted of homogeneously dispersed carbon nano-tubes in a polydimethylsiloxane polymer matrix prepared by an optimized synthesis process for actively monitoring the contractions/relaxations of muscles in the arm. In 2021 as well, Zhao et al. [35] introduced a sign language gesture recognition system that can differentiate fine-grained finger movements using the photoplethysmography (PPG) and motion sensors. An accuracy of 98% was attained by their system when differentiating nine finger-level gestures in American Sign Language. In addition, many sensors that are not commonly used in our daily lives have also been applied for SLR, such as RF sensors [36,37] and thermal sensors [38,39].

However, most of the aforementioned research only extracted a limited number of features from the raw data, which are not enough to fully exploit the potential capabilities of recognition models, especially for deep learning models. Little research has been conducted on improving the accuracy of CSLR by extracting multiple features from raw data to provide a diverse range of information to the underlying BLSTM model. To fill this research gap, we propose a pioneering BLSTM-based multi-feature framework, which extracts three sets of features from three different domains as the input features for the next BLSTM layer.

In addition, although many existing works have reached decent performance in terms of recognition accuracy, most of them either remain at a theoretical level, or only support recognition for digits and letters, which are still far away from real-world communication. To address this issue, we further develop a portable sign language collection and translation platform using the proposed BLSTM-based multi-feature framework to translate continuous sign language in real time to facilitate communication between deaf people and others.

## 3. The BLSTM-Based and Multi-Feature Framework

**Figure 1:**
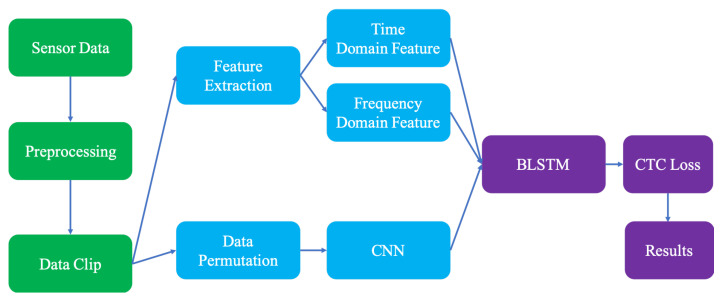
The structure of the proposed BLSTM-based multi-feature framework.

To minimize intrusions to signers and expand the application scenarios of this research, we adopted smart watches to conduct gesture-based CSLR in this work. Compared with vision-based CSLR and other wearable devices, only two types of activity-related data are provided by smart watches: accelerometer data and gyroscope data. This may lead to insufficient information provided to the recognition model and reduce the accuracy. To address this challenge, we propose a BLSTM-based multi-feature framework to conduct gesture-based CSLR accurately. Figure 1 demonstrates the structure of this framework in which three kinds of features are extracted from the preprocessed data, including time domain features, frequency domain features, and CNN-based features, and fed into the underlying BLSTM layer for recognition. In the rest of this section, this framework will be introduced in detail.

### 3.1. Preprocessing the Raw Data

Each data sample collected from the smart watches can be represented as $x^{T\times 12}$, in which *T* is the sequence length and 12 represents the dimensions of the 3-axis accelerometer and 3-axis gyroscope data of both hands. Before feeding the data into the proposed framework, a moving average filter with size 5 is firstly adopted to remove the noise from the accelerometer and gyroscope data. Then, a sliding window with size *L* and stride *S* is applied to convert the sensor data into *K* data clips, denoted as $X^{K\times L\times 12}$, in which *K* can be formulated as

(1) $$K=\frac{T-L}{S}+1.$$

### 3.2. Extracting the Time Domain and Frequency Domain Features

After the preprocessing, a total of 52 time domain features and 312 frequency domain features are extracted from each data clip. All the extracted time domain and frequency domain features are listed in Table 1. Basically, for time domain, five types of features are selected, including the mean, variance, magnitude of mean, covariance, and correlation of both accelerometer and gyroscope data. On the other hand, the intensities of each data column (12 columns in total) at the frequencies from 0 Hz to 25 Hz are considered as the frequency domain features. Thus, there are 312 frequency domain features in total. Compared with the time domain, we extract more features from the frequency domain to provide a wider range of spectrum information to the recognition model and improve the recognition accuracy.

### 3.3. Extracting the CNN-Based Features

**Figure 2:**
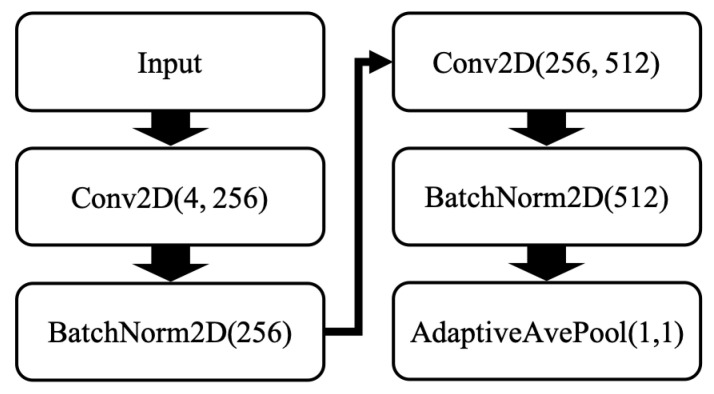
The CNN structure in the proposed framework.

In addition to time domain and feature domain features, the proposed framework also applies a convolutional neural network (CNN) to extract some trainable deep learning-based features from the sensor data. As one of the most successful network structures, CNNs have been widely used in solving different computer vision tasks [40]. Given enough training, CNNs can automatically discover the most important features from raw data, which is a desired ability for conducting gesture-based CSLR. However, the input for CNNs is usually a 3D matrix representing an image, while each preprocessed data clip in this framework has only two dimensions (L×12). To address this problem, each data clip is permuted from shape L×12 to L×4×3, in which 4 denotes the number of the sensors, including the $accelerometer_{left}$, $gyroscope_{left}$, $accelerometer_{right}$, and $gyroscope_{right}$, and 3 denotes the 3-axis data collected from each sensor. As shown in Figure 2, the CNN structure adopted in this framework is relatively shallow, including two 2D convolution layers followed by two batch normalization layers and one adaptive average pooling layer. The output channel of the last convolutional layer is designed to be 512. Thus, the size of the CNN-based features is also 512.

### 3.4. Sequential Learning with the BLSTM

After the feature extraction, we adopt the bidirectional long short-term memory (BLSTM) model to consider the temporal dependencies among the extracted features in this framework. Basically, the BLSTM is an updated version of LSTM [41] that allows the unit to obtain information from both its past units and its future units simultaneously. Just like LSTM, each BLSTM unit is composed of a cell, an input gate, an output gate, and a forget gate. The cell can store different values over arbitrary time intervals, while the three gates control the flow of information into and out of the cell. Because of its strong power in sequential learning, BLSTM has become one of the most popular approaches in solving various sequential data tasks, such as speech recognition [42] and natural language processing [43,44]. After the BLSTM layer, a fully-connected layer and a softmax layer are utilized to project the output of the BLSTM layer from feature space into vocabulary space and generate the results.

### 3.5. Framework Formulation

The formulations for the proposed BLSTM-based multi-feature framework can be explained as follows. Denoting the preprocessed data clips as $X^{K\times L\times 12}$ and the data clips after permutation as $X^{K\times L\times 4\times 3}$, three types of features are then extracted from these *K* data clips. According to Table 1 and Figure 2, there are 52 time domain features, 312 frequency domains features, and 512 CNN-based features, which can be represented as

(2) $$F_{Time}^{K\times 52}=f_{time}\left(X^{K\times L\times 12}\right),$$

(3) $$F_{Frequency}^{K\times 312}=f_{frequency}\left(X^{K\times L\times 12}\right),$$

(4) $$F_{CNN}^{K\times 512}=f_{cnn}\left(X^{K\times L\times 4\times 3}\right),$$

where $F_{Time},F_{Frequency}$, and $F_{CNN}$ represent the time domain features, frequency domain features, and CNN-based features, respectively. Then, all the extracted features are concatenated into one feature vector as the input for the following BLSTM layer:

(5) $$S^{K\times 2D}=f_{BLSTM}\left(concat\left(F_{Time},F_{Frequency},F_{CNN}\right)\right),$$

in which *D* is the hidden size and $S^{K\times 2D}$ is the output of the BLSTM layer. After that, the fully-connected layer projects the outputs of the BLSTM layer into vocabulary space and the last softmax layer generates the probability distribution accordingly, which can be denoted as

(6) P=softmax(W·S+b),

where *W* and *b* represent the weight matrix and the bias vector in the fully-connected layer, respectively.

### 3.6. Loss Function

As the ground truths in CSLR are sequences of words, connectionist temporal classification (CTC) [45] is utilized as the loss function in this framework. As one of the most commonly used solutions to address the alignment problem between the inputs and ground truth sequences, CTC introduces an extra “blank” token to denote the transition between two meaningful signs in the data stream. These “blank” tokens, together with the repeat tokens, are then be removed by the alignment process *V* to compute the CTC loss.

Assume P(s,t|x,θ) to be the probability of a specific sign *s* at time *t* computed by the proposed framework, where *x* and θ refer to the input sensor data and all the parameters in the proposed framework. Correspondingly, the probability of an arbitrary sentence $S=s_{t=1}^{T}$ can be represented as

(7) $$P\left(S|x;\theta \right)=\prod_{t=1}^{T}P\left(s_{t},t|x;\theta \right).$$

However, the sentence S will be considered as the correct recognition if and only if it satisfies the condition that V(S)=Y, where Y is the ground truth sentence. Therefore, the overall probability of all the correct recognitions can be computed as

(8) $$P\left(Y|x;\theta \right)=\sum_{V(S)=Y}P\left(S|x;\theta \right).$$

Given this probability of all the correct recognitions, the total CTC loss can then be computed as

(9) $$L_{CTC}\left(\theta \right)=-\log P\left(Y|x;\theta \right).$$

## 4. Experimental Results of the Proposed Platform on the HKSL Dataset

For this section, to demonstrate the effectiveness of the proposed BLSTM-based multi-feature framework, intensive experiments were conducted to compare it with other existing machine/deep learning approaches with a gesture-based continuous HKSL dataset, which was newly collected by us.

### 4.1. Information on the Proposed Continuous Sign Language Dataset

In this HKSL dataset, 50 commonly used sentences in the restaurants of Hong Kong are included. The English translations of these 50 sentences are listed in Table 2. Six signers performed each of these sentences eight times in Hong Kong sign language with two smart watches worn on both of their hands. During the signing, the hand movements of the signers were captured by the smart watches in the form of 3-axis accelerometer data and 3-axis gyroscope data, with a sample rate of 50 Hz. In addition, this dataset will continue to be updated so that more sentences can be included, and it will be opened to the public to facilitate research in gesture-based continuous sign language recognition.

### 4.2. Experimental Results

For this work, we conducted a large number of experiments to compare the performance of the proposed framework with other existing machine learning and deep learning approaches with the newly collected gesture-based continuous sign language dataset. In these experiments, word error rate (WER) was employed as the main criterion, which evaluates model performance based on the minimal operations (including substitution, deletion, and insertion) for converting the predicted sentences into the ground truth sentences. Assuming S,D, and *I* are the minimal requirements for substitutions, deletions, and insertions, the WER can then be formulated as

(10) $$\mathrm{WER}=\frac{S+D+I}{N},$$

where *N* is the number of words in the reference sentence. A lower WER represents better recognition performance. During the experiment, one signer was randomly selected as the testing signer, while other signers were considered as training data.

### 4.3. Comparison with Other Machine Learning Approaches

For this paper, five types of machine learning approaches [27,32,33,36,46] were selected for comparison, including the support vector machine (SVM), random forest (RF), K-nearest neighbors (KNN), linear discriminant analysis (LDA), and Gaussian mixture model (GMM).

Similar to the proposed BLSTM-based multi-feature framework, three types of features were extracted from the raw data and fed into these models during the experiment, including the time domain, frequency domain, and CNN-based features.

Table 3 presents the experimental results of the proposed framework and the machine learning methods. As can be observed from this table, the proposed framework achieved the lowest word error rate of 8.8%, which significantly outperforms the other machine learning methods by at least 13%. Among all the machine learning approaches, SVM reached the best performance with a word error rate of 22.7%. These experimental results strongly demonstrate the effectiveness of the proposed framework and the importance of adopting BLSTM to consider the sequential information in the gesture-based CSLR.

### 4.4. Comparison with Other Deep Learning Approaches

In the proposed multi-feature framework, three types of features are extracted from the sensor data: time domain, frequency domain, and CNN-based features. To study the performance and significance of these features, four types of deep learning methods were developed in this paper for comparison: “Time + BLSTM” [47], “Frequency + BLSTM” [30], “Time + Frequency + BLSTM” and “CNN + BLSTM” [29]. Except for the extracted features, most of the network structures of these methods remain the same as in the proposed BLSTM-based multi-feature framework. For instance, “CNN + BLSTM” represents that only CNN-based features are extracted and fed into the next BLSTM layer for recognition.

The experimental results of these deep learning methods are listed in Table 4. Compared with using only the time domain features or frequency domain features, a lower WER can be attained by combining the time domain and frequency domain features as the input features for the BLSTM layer. In addition, among the four deep learning approaches, “CNN + BLSTM” performed significantly better than the other methods, with a WER of 10.3%, which shows the effectiveness of the CNN-based features. More importantly, none of these four deep learning methods could outperform the proposed BLSTM-based multi-feature framework, which indicates the superiority of integrating multiple features in one framework for gesture-based CSLR.

## 5. The Portable Sign Language Collection and Translation Platform

**Figure 3:**
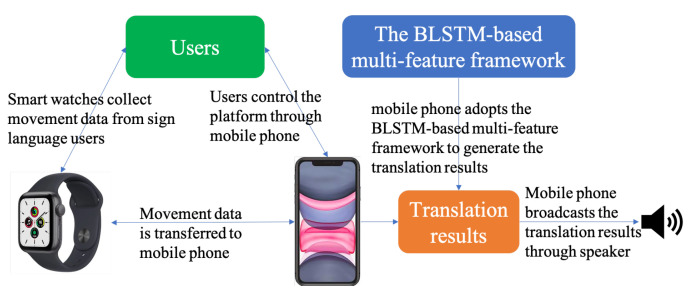
The structure of the portable sign language collection and translation platform.

To break down the communication barrier between sign language users and hearing people in a practical manner, we further developed a portable sign language collection and translation platform based on the BLSTM-based multi-feature framework. As shown in Figure 3, there are two major components in this platform: smart watches and a mobile phone, which are connected with each other through Bluetooth. The smart watches are used for measuring the hand movements of the signers through accelerometers and gyroscopes, while the mobile phone can manage the collected data and conduct CSLR. After that, the translation results can be broadcast by the speaker such that hearing people can understand the meaning of the sign language users

**Figure 4:**
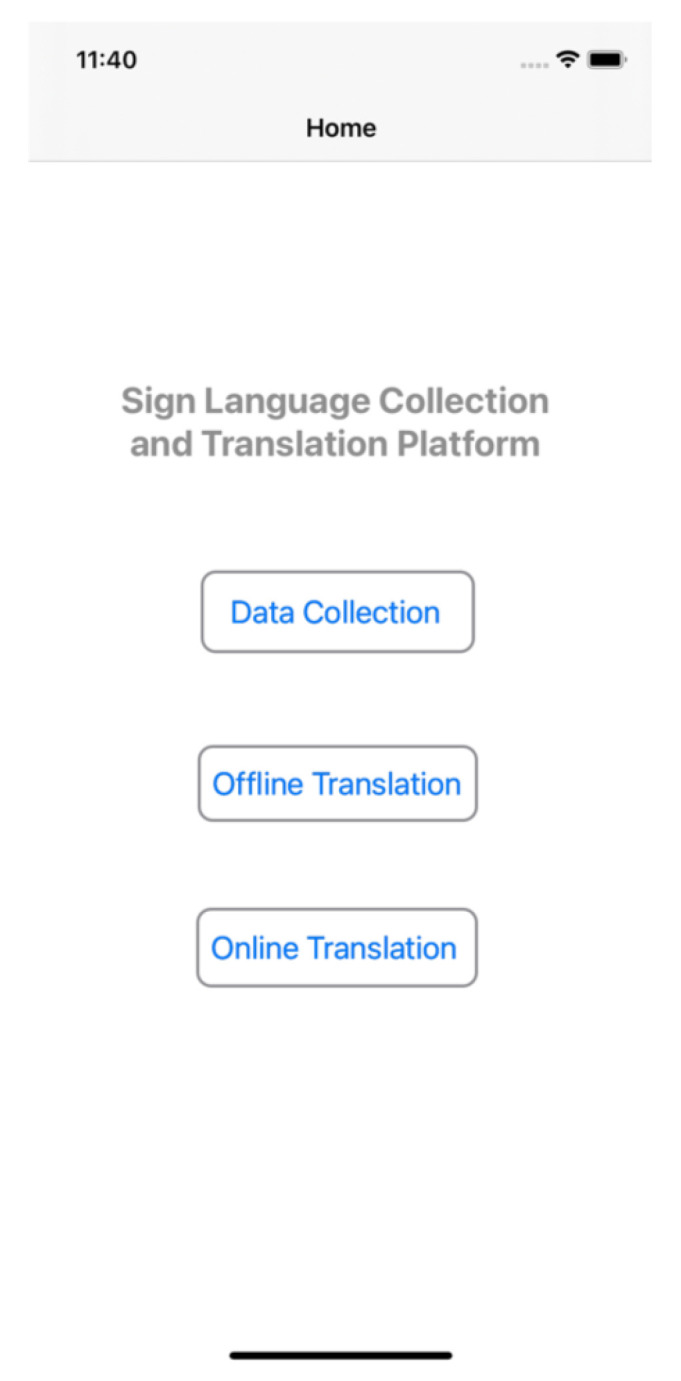
Three major systems in the proposed platform.

Figure 4 displays the interface of this platform, in which there are three main systems: the sign language dataset collection system, the offline sign language translation system, and the online sign language translation system. The functionalities and implementations of these systems will be introduced in this section.

### 5.1. The Sign Language Dataset Collection System

**Figure 5:**
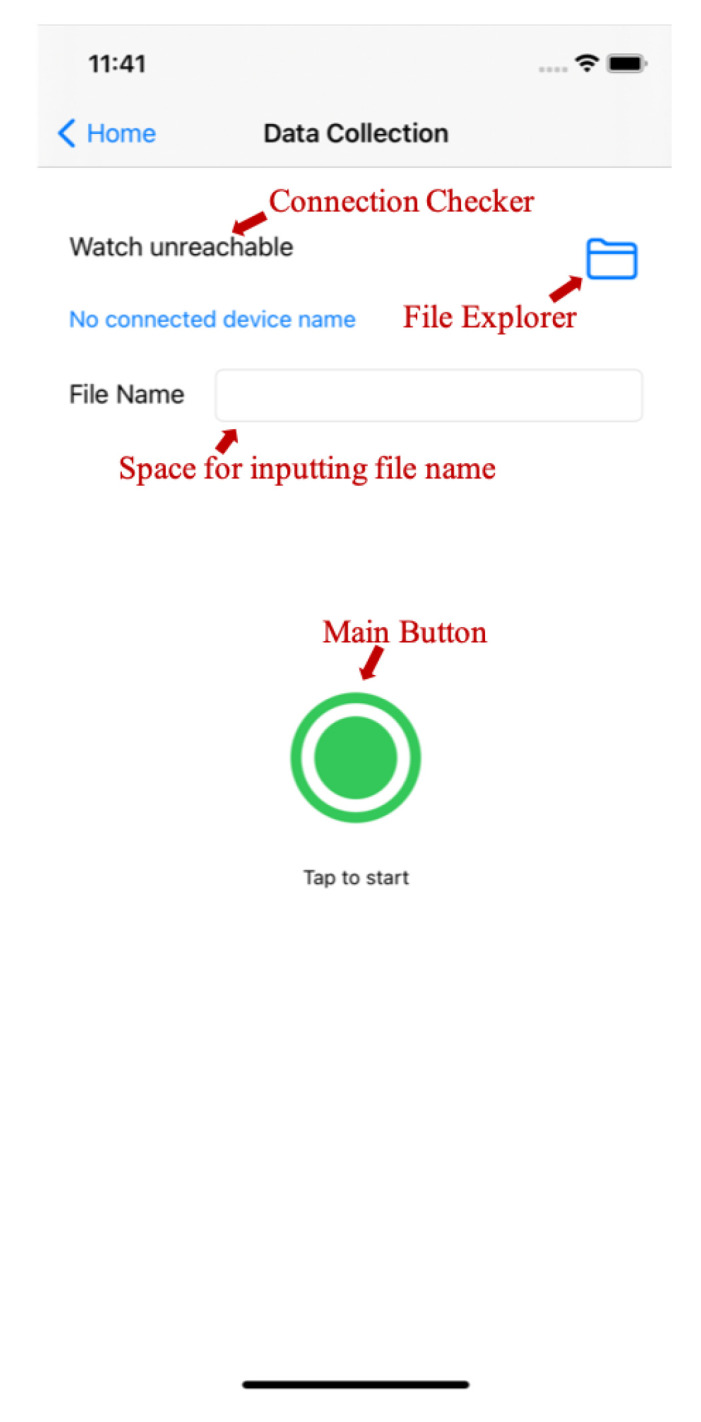
The dataset collection system.

Figure 5 shows the control panel of the sign language dataset collection system. Basically, there are four elements in this control panel: a connection checker, a file explorer, a space for inputting the file name, and a main button.

To use this system, the smart watches must firstly be connected to the mobile phone through Bluetooth, which will turn the connection checker from unreachable to reachable. Then, users can click the main button to start recording when the signer is ready, and click it again to stop recording. The sensor data collected from both the left hand and right hand are merged according to their timestamps and saved into one data file with the inputted file name after long pressing the main button. Each data file contains twelve data columns, including six columns for accelerometer data and six columns for gyroscope data (including both left hand and right hand). All the saved files can then be managed in the file explorer and be used to train the proposed BLSTM-based multi-feature framework.

### 5.2. The Offline Sign Language Translation System

**Figure 6:**
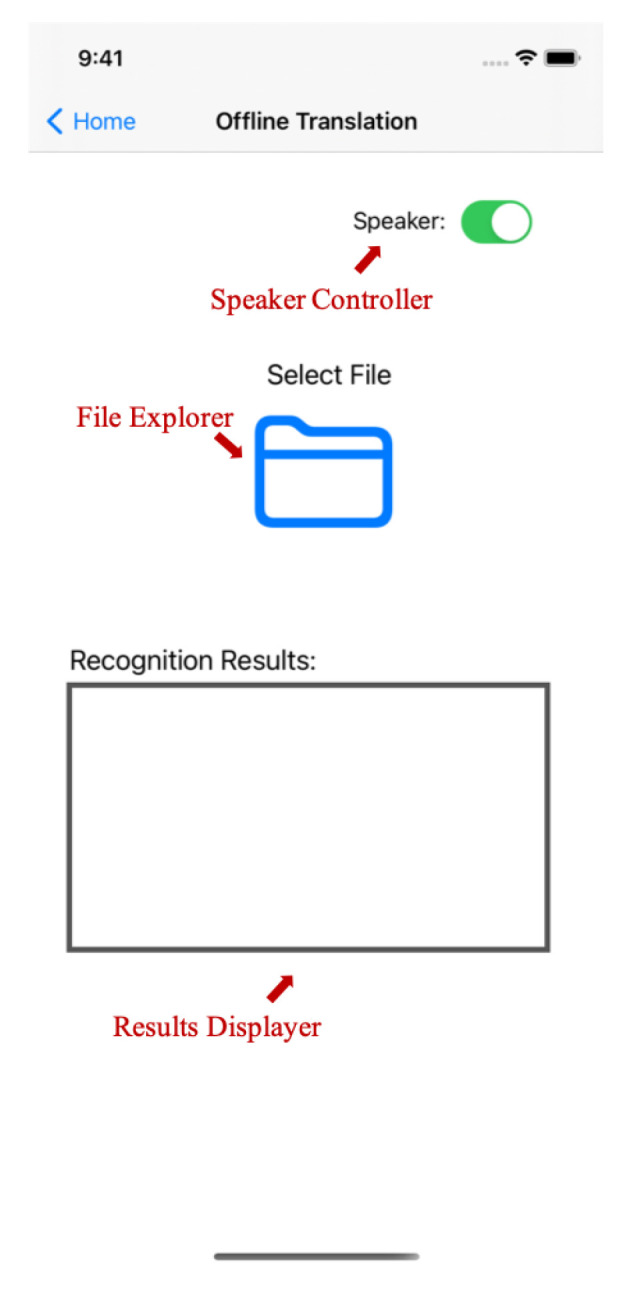
The offline translation system.

Figure 6 exhibits the interface of the offline sign language translation system. In this system, users can explore all the saved data files in the mobile phone with the file explorer and select one for translation. Given the selected data file, the BLSTM-based multi-feature framework introduced in Section 3 will be applied to translate the sign language into spoken language. The translation results will be shown in the window of the results displayer and then broadcasted by the mobile phone if the speaker is on. With this system, sign language users can record the most commonly-used sign language sentences in advance and broadcast their translations directly during real-world communication.

### 5.3. The Online Sign Language Translation System

**Figure 7:**
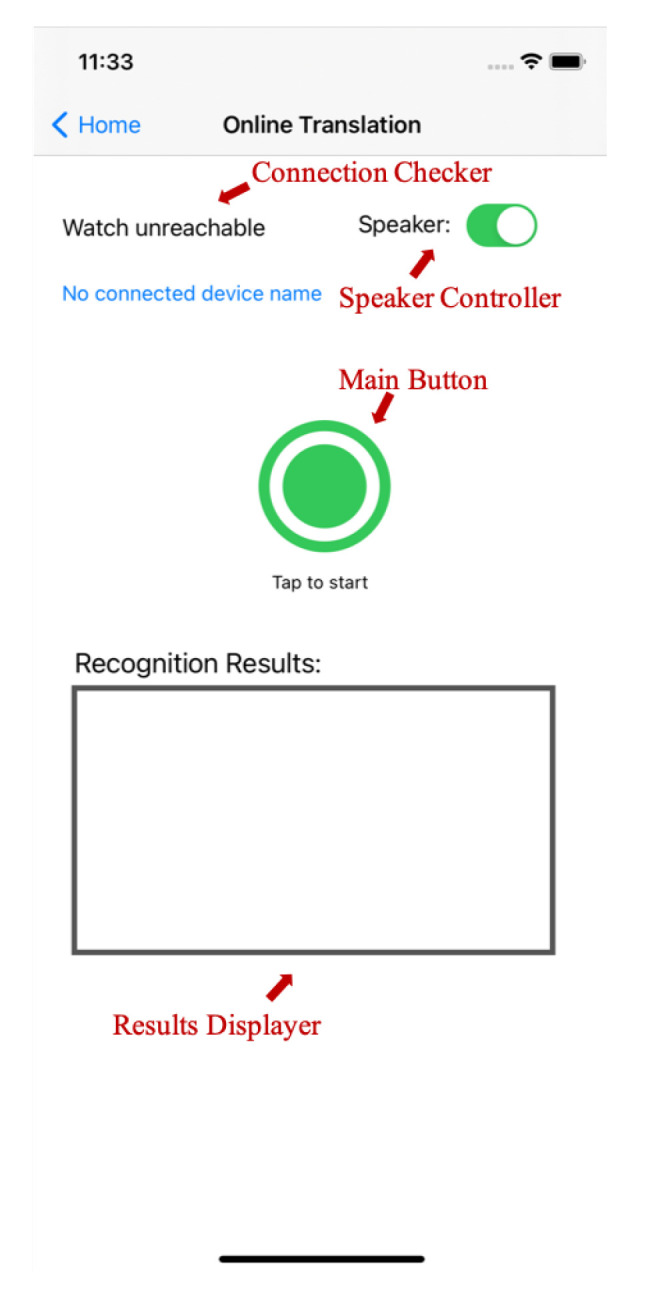
The online translation system.

The offline sign language translation system can only support the translation of the saved files, which is not convenient for instant communication. Therefore, we further introduce an online sign language translation system that can analyze the smart watch data and generate the translation results in real time. As shown in Figure 7, the interface of the online sign language translation system is similar to the sign language dataset collection system, in which there is a connection checker, a main button, a results displayer, and a speaker controller. To use this system, users should firstly wear the smart watches and ensure that the connection checker is showing as reachable, which indicates a stable Bluetooth connection between the smart watches and the mobile phone. Then, users can start to perform sign language after clicking the main button, and their hand movements will be recorded by the smart watches in the format of accelerometer and gyroscope data. After that, the proposed BLSTM-based multi-feature framework is employed to analyze the collected smart watch data. As it is online translation, the data continues to update during translation. Thus, this system will re-analyze all the collected data and re-generate a new translation result every $T_{1}$ seconds. If the data remain unchanged for more than $T_{2}$ seconds, it will be considered as the end of one sentence and the system will not combine the previous data before it with the latest data during the translation for next sentence. Both $T_{1}$ and $T_{2}$ are set to 1 in this system. In addition, when encountering the end of a sentence, the translation results of this sentence will be broadcast by the speaker of the mobile phone if the speaker controller is turned on, such that hearing people can understand the sign language performed by deaf people.

### 5.4. Experimental Results of the Proposed Platform

To demonstrate the effectiveness of the proposed platform, we conducted a user experiment to test its performance in translation accuracy and translation delay. In this experiment, we randomly selected 50 data samples from the testing set of the HKSL dataset and utilized the offline sign language translation system in the proposed platform to generate their translations. Table 5 lists the experimental settings and the experimental results. The proposed platform reached a WER of only 9.4% in this experiment, which is quite similar to the experiment results of the BLSTM-based multi-feature framework in Section 4. In terms of translation delay, an average translation delay of only 1.1 s was attained by the proposed platform, to generate the translations with a maximum delay of 1.5 s and a minimum delay of 0.8 s, which is short enough for real-time communication and translation. These experimental results strongly show the effectiveness of the proposed platform.

## 6. Concluding Remarks

Continuous sign language recognition (CSLR), which aims to recognize sequences of sign language from data generated from different types of sensors, is one of the most challenging, yet meaningful research directions in the area of accessible computing. Existing research works in CSLR focus on recognizing sign language from vision data, which is difficult to be apply in real-world translation, as it is severely limited by environmental conditions and computational resources. On the contrary, gesture-based CSLR does not suffer from these issues. By utilizing the sensor data provided by wearable devices, gesture-based CSLR can reduce translation delays significantly and support instant translation. However, it may cause imprecise CSLR due to the insufficient amount of information provided to the machine or deep learning models from the sensor data. To deal with this issue, we proposed an innovative BLSTM-based multi-feature framework, which extracts the time domain, frequency domain, and CNN-based features from the raw data as the input of the BLSTM layer for gesture-based CSLR. The experimental results on a newly collected gesture-based Hong Kong sign language dataset demonstrate that the proposed BLSTM-based multi-feature framework attains a lower word error rate (WER) in CSLR compared to other machine learning and deep learning approaches. To enable the integration of sign language users into the normal society, we further developed a portable sign language collection and translation platform with smart watches based on the proposed platform, which provides the functionalities of sign language collection and sign language translation.

More importantly, this work opens up numerous possible directions for future investigation. First, it is valuable to extract more features from sensor data to achieve higher accuracy of CSLR. Second, although CNN and LSTM are the most commonly used models in deep learning, there are still many other models such as the ResNet [9] and BERT [48] that may be explored for CSLR. Last but not least, except for mobile phones, other edge computing devices such as the Jetson Nano can also be employed in sign language collection and translation platforms [49] to shorten translation delays.

## Figures and Tables

**Table 1:** The time domain and frequency domain features extracted in the proposed framework.

	Feature Name	Feature Number
Time Domain	Mean	12
	Magnitude of Mean	4
	Variance	12
	Correlation	12
	Covariance	12
Frequency Domain	Intensities of the 12 columns at frequencies from 0 Hz to 25 Hz	312

**Table 2:** The 50 sentences in the proposed gesture-based continuous sign language dataset.

Number	English Translation	Number	English Translation
1	I ate a French toast	26	My sister ate two rices with pork
2	You ate two French toasts	27	My sister ate three rices with mutton
3	He ate three French toasts	28	My elder brother wants a spoon
4	We like pineapple bread	29	My elder brother wants two bowls
5	You like pineapple bread	30	My elder brother wants three chopsticks
6	They like pineapple bread	31	My elder sister wants a bowl
7	I don’t like sandwich	32	My elder sister wants two chopsticks
8	You don’t like sandwich	33	My elder sister wants three spoons
9	He doesn’t like sandwich	34	My brother wants a chopstick
10	I want three rices with barbecued pork	35	My brother wants two spoons
11	You want one rice with roast goose	36	My brother wants three bowls
12	He wants two rices with pork chop	37	I want a cup
13	I like rice with roast goose	38	You want two saucers
14	You like rice with pork chop	39	He wants three forks
15	He likes rice with barbecued pork	40	We want a saucer
16	We don’t like rice with pork chop	41	You want two forks
17	You don’t like rice with barbecued pork	42	They want three cups
18	He doesn’t like rice with roast goose	43	My father wants one fork
19	My mother wants a porridge with beef	44	My mother wants two cups
20	My mother wants two porridges with pork	45	My elder sister wants three saucers
21	My mother wants three porridges with mutton	46	My sister wants three cups of ice cola
22	My father doesn’t like soup with beef	47	My grandfather wants two cups of ice cola
23	My father doesn’t like soup with pork	48	My grandmother wants one cups of ice cola
24	My father doesn’t like soup with mutton	49	My grandfather doesn’t like ice water
25	My sister ate a rice with beef	50	My grandmother doesn’t like ice water

**Table 3:** The experimental results of the machine learning approaches and the proposed framework.

Method	WER
Time + Frequency + CNN + SVM	0.227
Time + Frequency + CNN + RF	0.249
Time + Frequency + CNN + KNN	0.251
Time + Frequency + CNN + LDA	0.258
Time + Frequency + CNN + GMM	0.378
The Proposed BLSTM-Based and Multi-Feature Framework	0.088

**Table 4:** The experimental results of the existing deep learning approaches and the proposed multi-feature framework.

Method	WER
Time + BLSTM	0.208
Frequency + BLSTM	0.232
Time + Frequency + BLSTM	0.167
CNN + BLSTM	0.103
The Proposed BLSTM-Based and Multi-Feature Framework	0.088

**Table 5:** Experimental results of the proposed platform.

Number of Data Samples	50	Mobile Phone Model	iPhone XR
Maximum Translation Delay	1.5 s	Minimum Translation Delay	0.8 s
Average Translation WER	9.2%	Average Translation Delay	1.1 s
